# Low rate of early periprosthetic fractures in cementless short-stem total hip arthroplasty using a minimally invasive anterolateral approach

**DOI:** 10.1186/s10195-021-00583-x

**Published:** 2021-05-21

**Authors:** Matthias Luger, Günter Hipmair, Clemens Schopper, Bernhard Schauer, Rainer Hochgatterer, Jakob Allerstorfer, Tobias Gotterbarm, Antonio Klasan

**Affiliations:** 1 Department for Orthopedics and Traumatology, Kepler University Hospital GmbH, Krankenhausstrasse 9, 4020 Linz, Austria; 2 Johannes Kepler University Linz, Altenberger Strasse 69, 4040 Linz, Austria

**Keywords:** Short stem, Total hip arthroplasty, Minimally invasive, Anterolateral approach, Watson-Jones, Periprosthetic fracture

## Abstract

**Purpose:**

Minimally invasive (MIS) approaches in combination with short stems have gained popularity in recent years in total hip arthroplasty (THA). A decreased risk for periprosthetic femoral fractures (PFFs) is reported for cementless short-stem THA, but in contrast to other approaches, the risk factors for PFFs for short-stem THA using MIS anterolateral approach in supine position are not described in literature.

**Methods:**

A single-center consecutive series of 1052 hips in 982 patients, performed between 2014 and 2019 with a short curved stem and a press fit using an MIS anterolateral approach in supine position, was retrospectively screened for inclusion. Fourteen patients were lost to follow-up. Therefore, 1038 THAs in 968 patients were included. Risk factors for intra- and postoperative PFFs within 90 days were analyzed. We investigated for sex, age, body mass index (BMI), diagnosis, and laterality.

**Results:**

In total, 18 PFFs (1.7%) occurred. Intraoperative fracture occurred in ten cases ( 0.9%), with another eight cases (0.8%) occurring postoperatively. Increased American Society of Anesthesiologists (ASA) Score was a significant risk factor for PFF (*p* = 0.026), whereas sex (*p* = 0.155), age (*p* = 0.161), BMI (*p* = 0.996), and laterality (*p* = 1.000) were not. Seven PFFs (0.7%) required revision arthroplasty.

**Conclusion:**

Cementless short-stem THA using the MIS anterolateral approach is a procedure with a low number of PFFs within 90 days from index surgery. Fracture rates are comparable to other MIS approaches, and comparable femoral short stems are used. Age, sex, and BMI were not identified as risk factors of PFF, while risk for PFF increased with ASA Score.

**Level of Evidence:**

Level IV

Periprosthetic femoral fracture (PFF) is a major complication in total hip arthroplasty (THA). The overall mortality risk within 12 months of the complication increases up to 11% in patients with PFF [1, 2]. Additionally, PFF often requires complex revision surgery [3, 4], which increases postoperative readmission and functional limitation after revision surgery [2, 5]. The prevalence of PFFs appears to be increasing [5, 6], with an expected increase of 4.6% every decade over the next 30 years [7].

Minimally invasive (MIS) approaches in THA have gained popularity over the last years owing to faster recovery, less pain, and fewer postoperative precautions [8, 9]. In particular, anterior-based MIS approaches are increasingly performed for THA, including the direct anterior approach (DAA) as well as the abductor-sparing MIS Watson-Jones anterolateral approach [10]. With the popularization of MIS approaches, femoral short stems are utilized more frequently in THA as they facilitate soft-tissue sparing implantation [11].

Periprosthetic fractures (PFFs) are a known risk factor in anterior approaches [8–10]. The rate of PFFs in MIS anterolateral approach can be as high as 8.3% within 90 days from index surgery [10]. Especially fractures of the greater trochanter are described in the literature, with a rate of 3.2% in nonobese and up to 9.7% in obese patients [9].

Female sex [12, 13], increasing age [12–15], presence of osteoporosis [15], and rheumatoid arthritis have been associated with increasing rates of PFFs [5]. In particular, cementless femoral components have been associated with a higher risk of intra- and postoperative PFFs [15–18]. In a recent review, cementless femoral implants in general, and especially single-wedge and double-wedge components, have higher rates of PFFs [5]. Cementless short-stem THA shows a reduced risk for PFFs compared with standard cementless straight stems [19]. The rate of PFFs was found to be significantly decreased in short stem in DAA, with 1.6% compared with 6.8% in cementless straight stems [19]. Molli et al. [20] proposed that a short stem could decrease the incidence of intraoperative periprosthetic fracture compared with a standard-length stem because of less load during broaching.

The rate of PFFs in cementless short-stem arthroplasty is not fully evaluated, especially in minimally invasive anterolateral approach. Therefore, we conducted this study to evaluate the rate of early PFFs in cementless short-stem THA using an MIS anterolateral approach in supine position.

## Methods

### Patients

A retrospective evaluation of consecutive THAs of a single center performed via a minimally invasive anterolateral approach using a cementless, curved short stem (Fitmore stem, ZimmerBiomet, Warsaw, IN, USA) and cementless titanium press-fit cup with or without screws (Allofit/-S, ZimmerBiomet, Warsaw, IN, USA) was carried out. The study was approved by the institutional review board (EK-No.: 1239/2019) in accordance with the World Medical Association Declaration of Helsinki. Because it was a retrospective evaluation of preexisting medical records, informed consent was not required.

A consecutive series of 1052 hips in 982 patients with index surgery between 2014 and 2019 was analyzed, and the medical records until 90 days postoperation were evaluated. Fourteen patients were lost to follow-up. Consequently, 1038 hips in 968 patients were included. All electronically saved and archived medical records were reviewed, including operative reports, postoperative notes, discharge summaries, and postoperative medical records. Age, sex, weight, height, body mass index (BMI), American Society of Anesthesiologists (ASA) Score, preoperative diagnosis, and laterality were documented. All reports of intra- and postoperative fractures within a 90-day postoperative period were collected.

### Surgical technique and treatment protocol

Surgical procedures were carried out at the author’s institution by surgeons with different levels of experience including 11 consultants and 7 residents. All consultants perform more than 50 and all senior consultants more than 100 arthroplasties per year. Resident surgeries were done under the guidance of a consultant. In all cases, a minimally invasive anterolateral Watson-Jones approach in supine position on a standard operating table under laminar flow was performed [21]. Extremity preparation was performed with threefold antiseptic scrub with alcohol disinfectant. Draping with sterile adhesive surgical iodine film was used. A skin incision was centered over the greater trochanter. An incision at the border between the tensor fasciae latae and the tractus iliotibialis was performed. Then, the Watson-Jones interval between tensor fasciae latae and gluteus medius was bluntly dissected. A capsulectomy was performed in each case. The standardized peri- and postoperative protocol was identical in all cases, including single-shot antibiotics [cefuroxime 1.5 g intravenous (i.v.), directly preoperatively], weight-bearing as tolerated from the first postoperative day on, indomethacin 75 mg twice daily for the prevention of heterotopic ossification on days 1–4 postoperatively, and 40 mg low-molecular-weight heparin or rivaroxaban 10 mg for 28 days postoperatively as venous thromboembolic event prophylaxis.

In all patients, a cementless, curved short stem (Fitmore stem, ZimmerBiomet, Warsaw, IN, USA) was digitally templated using mediCAD version 5.1 (Hectec GmbH, Altdorf, Germany). Fitmore hip stem is a titanium alloy stem (Ti Al6V4) that has a Porolock Ti-VPS coating in the proximal part to enhance bone ingrowth and is available in four different neck angle options (127°, 129°, 137°, 140°) [22]. A cementless titanium press-fit cup with or without screws (Allofit/-S, ZimmerBiomet, Warsaw, IN, USA) was used in all patients.

In the case of suspected or apparent intraoperative PFF, fluoroscopy was draped and utilized. Fractures of the greater trochanter were treated either nonoperatively or with a cerclage wire, depending on the stability of the fracture and stem. Each patient was mobilized with touch weight-bearing for 6 weeks. In the case of intraoperative fractures of the calcar, medial, or lateral cortex, we performed a reduction around the implanted stem using cerclage wires. In the case of primary stability, Fitmore hip stem was kept in situ. Patients were mobilized with touch weight-bearing for 4 weeks. If primary stability was not achieved, a cementless straight stem (Alloclassic SL, ZimmerBiomet, Warsaw, IN, USA) or a cementless monoblock revision straight stem (Alloclassic SLL, ZimmerBiomet, Warsaw, IN, USA) was used. In the case of stem revision, patients were mobilized with touch weight-bearing for 4 weeks. In the case of intra- or postoperative PFF, patients received a clinical and radiological follow-up prior to permission of full weight-bearing. Patients were then followed clinically and radiologically at our outpatient department 3 months and 1 year after occurrence of PFF.

### Statistics

Descriptive statistical analysis was conducted for age, sex, body mass index (BMI), preoperative diagnosis, and laterality. Shapiro–Wilk test was performed for testing for normality distribution. As not all variables were normally distributed, nonparametric testing was performed. Fisher’s exact test was performed for categorical variables (sex, diagnosis, ASA Score, laterality) to evaluate any association between independent variables and likelihood of a fracture. Post-hoc calculations with Bonferroni correction were carried out in the case of significant differences. Wilcoxon Mann–Whitney *U* test was performed on continuous variables (age and BMI). Binary logistic regression models for dichotomous outcomes were estimated to model the effect of sex, age, BMI, surgeon’s experience, and ASA Score on the likelihood of a fracture. A power analysis was not performed owing to the consecutive recruitment of patients over a longer time period with an observed PFF rate [23]. Statistical analysis was calculated with SPSS version 26 (IBM SPSS statistics, Chicago, IL, USA). A* p* value < 0.05 was considered as statistically significant.

## Results

In total, 1038 hips in 968 patients were included. Eighteen (1.7%) periprosthetic fractures (PFFs) occurred. Of these fractures, ten PFFs (0.9%) occurred intraoperatively, while eight PFFs (0.8%) occurred postoperatively within the first 90 days after index surgery. Thirteen cases (1.25%) of PFFs occurred in female patients, and five in male (0.45%) patients. Average age at operation was 67.24 years (range 24.21–99.02 years), in patients without PFF 67.23 years (range 24.21–94.38 years), and in patients with PFF 71.78 years (range 42.04–99.02 years). Average time to diagnosis of postoperative fracture was 18.75 days (range 4–70 days). Average BMI was 27.92 ± 4.96 kg/m^2^ (range 16.38–48.93 kg/m^2^), in patients with PFF 28.0 ± 5.62 kg/m^2^ (range 17.78–43.44 kg/m^2^), and in patients without PFF 27.91 ± 4.95 kg/m^2^ (range 16.38–48.93 kg/m^2^). Revision rate was 0.7%, with 7 of 18 PFFs needing revision arthroplasty. Full patient demographics are presented in Table 1.

**Table 1:** Patient demographics and *p* values for testing for statistical significance of occurrence in PFF stratified for sex, age, diagnosis, ASA Score, laterality, and BMI

Patient demographics		*p* Value^a^
Sex, *n* (%)		0.155
Male	472 (45.5%)	
Female	566 (54.5%)	
Age (years)	67.24 ± 11.37 (24.21–99.02)	0.161
Average age (no fracture)	67.23 ± 11.32 (24.21–94.38)	
Average age (fracture)	71.78 ± 13.77 (42.04–99.02)	
Diagnosis, *n* (%)		0.005
Primary osteoarthritis	878 (84.6%)	
Avascular necrosis	97 (9.3%)	
Hip dysplasia	47 (4.5%)	
Femoral neck fracture	2 (0.2%)	
Posttraumatic osteoarthritis	14 (1.3%)	
ASA Score, *n* (%)		**0.026**
ASA I	195 (18.8%)	
ASA II	591 (56.9%)	
ASA III	**247 (23.8%)**	
ASA IV	5 (0.5%)	
Laterality, * n* (%)		1.000
Left	488 (47.0%)	
Right	550 (53.0%)	
BMI (kg/m^2^)	27.92 ± 4.96(16.38–48.93)	0.996
BMI (no fracture)	27.91 ± 4.95(16.38–48.93)	
BMI (fracture)	28.0 ± 5.62(17.78–43.44)	
Surgeon’s experience (*n*, THAs)		
Consultants	788 (75.9%)	
Residents	250 (24.1%)	
Fractures		
Total	18 (1.7%)	
Female	13 (1.25%)	
Male	5 (0.45%)	
Intraoperative fractures	10 (0.9%)	
Postoperative fractures	8 (0.8%)	
Revisions	7 (0.7%)	
Fractures CCD angle	Fractures/THAs	0.648
A (140°)	4/186 (2.2%)	
B (137°)	12/604 (2.0%)	
B extended (129°)	2/244 (0.8%)	
C (127°)	0/18 (0.0%)	

^a^Testing patients with fracture versus patients without fracture

**Fig. 1:**
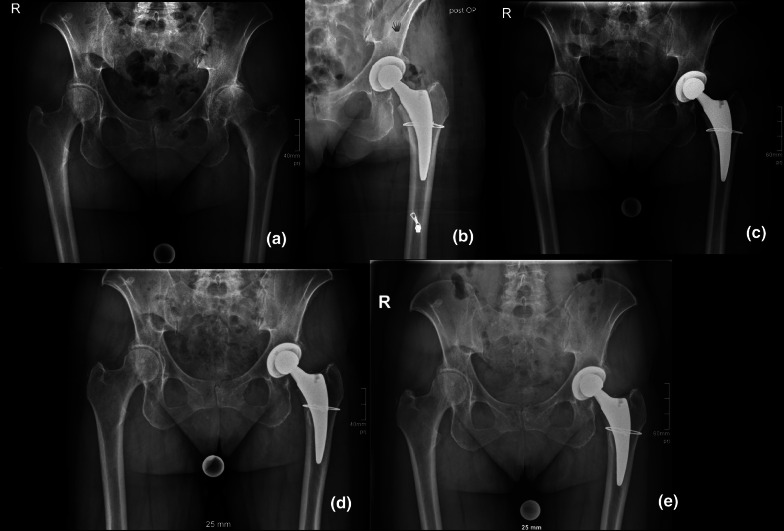
Intraoperative fracture of the calcar treated with one cerclage wire: **a** preoperative; **b** postoperative; **c** 6 weeks postoperative; **d** 3 months postoperative; **e** 1 year postoperative

**Fig. 2:**
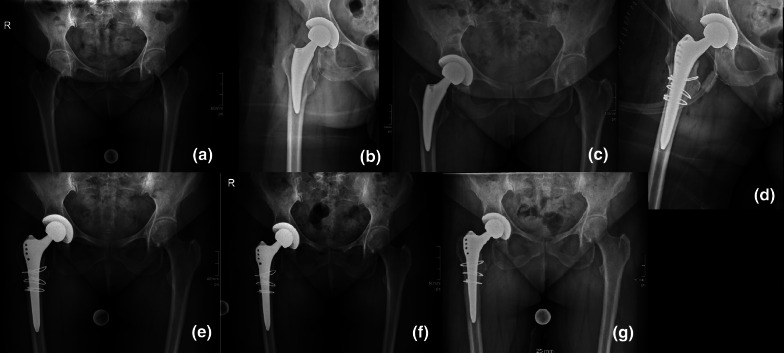
Occult fracture of the medial cortex detected on the fourth postoperative day; **a** preoperative; **b** postoperative; **c** fourth postoperative day; **d** postoperatively after revision (Alloclassic SL (ZimmerBiomet) with three cerclage wires); **e** 6 weeks after revision; **f** 3 months after revision; **g** 1 year after revision

**Fig. 3:**
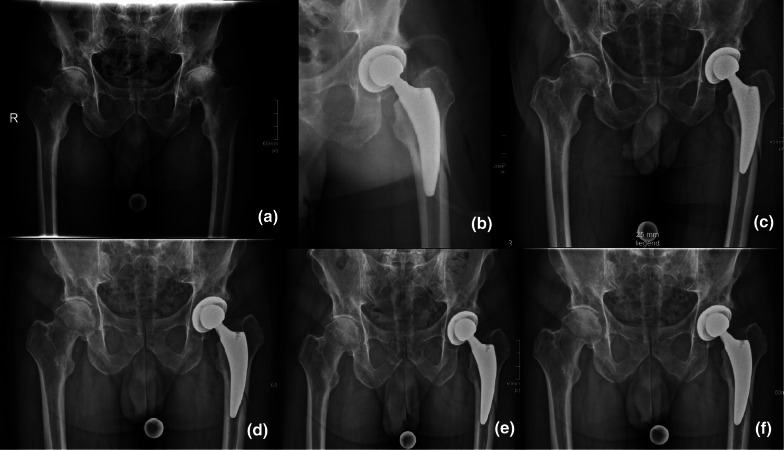
Intraoperative fracture treated without revision surgery; detected on day of surgery on postoperative x-ray with patient already outside the operating theater; patient did not give informed consent for revision surgery; conservative treatment with 6 weeks without weight-bearing; **a** preoperative, **b** postoperative, **c** discharge at home; **d** 6 weeks after surgery; **e** 3 months after surgery; **f** 1 year after surgery

Intraoperative fractures occurred in ten patients (55.6%), while eight PFFs (44.4%) were diagnosed postoperatively. Type of fracture and time of occurrence/diagnosis of PFF are presented in Table 2. Table 3 presents cause of fracture, time of fracture in days, Vancouver classification of postoperative PFF, and treatment for every PFF in detail. Figures 1, 2 and 3 show different PFFs and their treatment.

**Table 2:** Type of PFFs and time of occurrence/diagnosis

Type (*n*)	Intraoperatively	Postoperatively	Percentage of all fractures	Percentage of all hips
Medial cortex (*n* = 8)	5 (27.8%)	5 (27.8%)	55.6%	1.0%
Calcar (*n* = 1)	1 (5.6%)	0 (0.0%)	5.6%	0.1%
Lateral cortex (*n* = 3)	2 (11.1%)	2 (11.1%)	22.2%	0.4%
Greater trochanter (*n* = 3)	1 (5.6%)	2 (11.1%)	16.7%	0.3%

**Table 3:** Cause of fracture, time of fracture, Vancouver classification, and treatment for all PFFs

Fracture type	Cause of fracture	Time of fracture	Vancouver classification	Treatment
Intraoperatively (*n* = 10)				
Medial cortex	Implant insertion			One cerclage cable
Medial cortex	Implant insertion			One cerclage cable
Calcar	Implant insertion			One cerclage cable
Lateral cortex	Implant insertion			Two cerclage cables
Lateral cortex	Implant insertion			Conservatively, no weight-bearing for 6 weeks
Medial cortex	Broaching			One cerclage cable
Greater trochanter	Retraction with Hohmann retractor			One cerclage cable
Medial cortex	Implant insertion			One cerclage cable
Medial cortex	Implant insertion			Straight stem, two cerclage cables
Lateral cortex	Failed cerclage of intraoperatively detected fracture	Revision on day 31	Vc B2	Revision arthroplasty (monoblock revision stem, three cerclage cables)
Postoperatively (*n* = 8)				
Greater trochanter	Avulsion	9d	Vc A_g_	Conservatively
Medial cortex	No history of fall	6d	Vc B2	Revision arthroplasty (straight stem, four cerclage cables)
Medial cortex	No history of fall	4d	Vc B2	Revision arthroplasty (straight stem, three cerclage cables)
Medial cortex	No history of fall	14d	Vc B2	Revision arthroplasty (straight stem, three cerclage cables)
Medial cortex	Fell while walking	24d	Vc B2	Revision arthroplasty (Monoblock revision stem, three cerclage cables)
Medial cortex	No history of fall	14d	Vc B2	Revision arthroplasty (straight stem, three cerclage cables)
Greater trochanter	Avulsion	5d	Vc A_g_	Conservatively
Lateral cortex	Fell while walking	70d	Vc B2	Revision arthroplasty (monoblock revision stem, two cerclage cables)

### Testing and logistic regression

The detailed results for testing are presented in Table 1 and for logistic regression in Table 4. A significance was detected for ASA Score with *p* = 0.026. Post-hoc calculation showed a significance for group ASA Score III. Testing for influence of diagnosis on PFF showed a *p* value of 0.005 for osteoarthritis. However, post-hoc calculations did not show any significance. Logistic regression showed an increased risk of PFF occurrence depending on diagnosis. Logistic regression showed an increased risk for PFFs with increasing ASA Score. A statistical significance for age and sex in occurrence of PFFs could not be detected for either variable. Logistic regression did not show any significance or increased risk for age, sex, or BMI in occurrence of PFFs.

**Table 4:** Logistic regression for age, sex, BMI, ASA Score, diagnosis, surgeon’s experience, and neck angle; odds ratio, confidence interval (95%), and *p* value

	Odds ratio	Confidence interval	*p* Value
Age	1.041	0.994–1.090	0.089
Sex	0.455	0.161–1.287	0.138
BMI	1.003	0.914–1.102	0.944
ASA Score	**2.476**	**1.195–5.129**	**0.015**
Diagnosis	**1.679**	**1.009–2.984**	**0.046**
Surgeon’s experience	1,217	0.429–3.447	0.712
Neck angle	0.618	0.292–1.308	0.208

Significant value in bold letters

## Discussion

The use of cementless femoral short stems in MIS THA has increased in the last years. The rate of PFFs in cementless THA differs considerably, up to 27.8% [19]. Use of femoral short stems is described as decreasing the risk for PFF [19, 20, 24]. Dietrich et al. [19] investigated the rate of PFFs using DAA with short stems (including Fitmore short stem) in 183 THAs and found a significantly reduced rate of PFFs of 1.6% compared with 6.8% in 457 THAs with conventional straight stems. We report a comparable rate of 1.7% for MIS anterolateral approach. The use of short stems leads to reduction fractures of the greater trochanter [19]. Yu et al. [24] investigating the Tri-Lock stem (Depuy, Warsaw, IN, USA) in 103 patients found no intraoperative periprosthetic fracture in the short-stem group, compared with 8.6% with the conventional stem. For MIS anterolateral approach, the rate of greater trochanter fractures is reported to be between 2.5% and 3.2% in nonobese and up to 9.7% in obese patients [8–10]. Our results show a low rate of 0.3% PFFs of the greater trochanter. Fitmore hip stem can be inserted initially in a more varus oriented position, following a C-shaped path out of varus until it is ultimately oriented with the mechanical axis of the diaphysis [22]. Therefore, we postulate that short curved stems in MIS anterolateral approach reduce the pressure on the greater trochanter, leading to a reduced rate of fractures of the greater trochanter.

Age is also described as a risk factor for PFF, with a risk 2.9 times higher in patients over 70 years for all approaches [25]. Also, in DAA as well as MIS anterolateral approach an increased risk in older patients is described [10, 26, 27]. Berend et al. [26] report age as a risk factor for PFFs in DAA, with the average age of patients with fractures being 72 years compared with 63 years without fractures. Herndon et al. [10] did not find a significance for age (*p* = 0.13) but postulated age being a risk factor, because of a higher average age in the fracture group, with 69.2 years compared with 66.6 years in the nonfracture group. Gkagkalis et al. [11] report a similar rate for intraoperative fissures in short-stem arthroplasty in elderly patients (over 75 years) compared with a younger control group (< 60 years), with 1.5% and 1.4%, respectively. Our results show an average age in patients with fracture with 71.78 years compared with 67.23 years in patients without fracture. Age in patients with PFF is on average 4.55 years higher. However, we could not find a statistical significance (*p* = 0.161).

PFF occurred more often in female patients, with 13 PFFs compared with 5 PFFs in male patients. However, testing did not show significance (*p* = 0.155). Logistic regression found an OR of 0.455 (CI 0.161–1.287; *p* = 0.138). The odds ratio indicates a lower risk for occurrence of PFFs in male patients, but without statistical significance. Female sex is also associated with higher risk for PFFs [10, 27, 28]. The results in the presented study could not support these findings. We report a consecutive case series with over 1000 implantations with findings contrary to comparable studies [10, 27]. Sheth et al. [28] report an increased OR of 2.74 (CI 1.28–5.89; *p* = 0.01) for female sex in 5313 primary THAs with a high number of different stem designs and fixation. We report a higher number of PFFs occurring in female patients and a lower OR for male patients but cannot support these findings with statistical significance. Therefore, female sex could not be fully proven as a risk factor for PFF in cementless short-stem THA.

The presented study reports a revision rate of 0.7%, with 38.89% of all PFFs needing revision arthroplasty. Herndon et al. [10] report a revision rate of 2.0%, with 48,3% of all PFFs needing revision arthroplasty. Sheth et al. [28] report a revision rate of 100% of all 32 PFFs that occurred within 90 days after index surgery. Watts et al. [29] report revision arthroplasty in 50% and 76%, respectively, for two different stem designs, while Gromov et al. [30] report revision arthroplasty in 97.0% of all PFFs. In our study, PFFs could be fixed using a standard straight stem or its monoblock revision straight stem derivate in the case of revision arthroplasty. These findings indicate a major advantage of cementless short stems of preserving more bone stock, and a different fracture type in the case of PFF occurrence resulting in easier revision arthroplasty.

The type of fixation is also discussed broadly regarding the occurrence of PFFs. Cemented femoral components are associated with a significantly lower rate of PFFs in direct comparison with cementless stems [5, 28, 31]. However, indication for THA is inhomogeneous. In particular, cementless fixation in femoral neck fracture is associated with an increased rate of PFFs of up to 7.4% [31, 32], but yields better functional scores [31]. Fitmore short stem shows excellent outcome regarding aseptic loosening, with 99.6% survival rate after 8.6 years and excellent functional outcome with an average Harris Hip Score between 96 and 98 points after an average follow-up of 7.7 years [33]. Our results show a low number of PFFs without any statistical significance for age and sex. Therefore, cementless short-stem THA using minimally invasive approaches yields excellent functional outcome combined with a significantly reduced rate of PFFs compared with cementless straight-stem THA.

Some limitations need to be noted. Although this is a retrospective study of consecutive patients, selection bias cannot be excluded. Another limitation is the limited follow-up period of 90 days within index surgery, which is due to the retrospective study design. A longer follow-up period of 1 year might have resulted in a significantly higher number of patients lost to follow-up and might have impaired the number of included patients immensely. Therefore, we favored a significantly higher number of patients and implantations with a shorter follow-up period to prevent a high number of patients being lost to follow-up.

## Conclusion

Cementless total hip arthroplasty with a curved short stem using the minimally invasive anterolateral approach shows a low number of intra- and postoperative periprosthetic fractures within 90 days from index surgery. Fracture rates are comparable to other minimally invasive approaches and other comparable femoral short stems. Age and sex are not associated with a higher risk for periprosthetic fractures.

## Data Availability

Data and materials are available on request.
